# Successful nintedanib desensitization: Due to two cases

**DOI:** 10.5578/tt.20239912

**Published:** 2023-03-10

**Authors:** F.M. TEPETAM, Ş. ÖZDEN, D. DUMAN

**Affiliations:** 1 Clinic of Immunology and Allergy, Süreyyapaşa Chest Diseases and Chest Surgery Training and Research Hospital, İstanbul, Türkiye; 2 Clinic of Chest Diseases, Süreyyapaşa Chest Diseases and Chest Surgery Training and Research Hospital, İstanbul, Türkiye

Dear Editor

Idiopathic pulmonary fibrosis (IPF) is a chronic and progressive lung disease with high mortality. Nintedanib is one of two oral antifibrotic agents approved for use in the treatment of IPF. Nintedanib is an intracellular tyrosine kinase inhibitor that targets multiple receptors such as vascular endothelial growth factor (VEGF), fibroblast growth factor (FGF), and platelet-derived growth factor (PDGF) ( 1 , 2 ). The most common side effect is mild to moderate diarrhea. Only 5% of the patients have diarrhea that is severe enough to require discontinuation of treatment. Other common side effects have been reported as infections such as bronchitis, and nasopharyngitis and nonspecific symptoms such as weakness, vomiting, and cough ( 3 ). To the best of our knowledge, no drug hypersensitivity reaction has been reported with nintedanib. However, there are case reports of serious skin reactions after pirfenidone, another antifibrotic agent approved for the treatment of IPF. Adverse skin reactions after pirfenidone use were reported more frequently in the first six months of treatment ( 4 ). Dose reduction is recommended for such side effects. In the presence of symptoms that may indicate liver damage, such as fatigue, anorexia, right upper quadrant pain, or jaundice, after nintedanib use, discontinuation of treatment or dose reduction is recommended if aspartate aminotransferase (AST) or alanine aminotransferase (ALT) is more than three times the upper limit of normal, or if it is more than five times the normal limit in asymptomatic patients ( 5 ). However, in hypersensitivity reactions, when the patient is exposed to the drug even at low doses, more serious systemic reactions may develop than in the previous history. Since no allergic reactions with nintedanib have been reported in the literature, the approach in such patients is unclear. In general, in patients with a history of drug hypersensitivity reactions, the decision is made according to the severity of the reaction. If it is not a serious lifethreatening reaction such as anaphylaxis and it is essential for the patient to use this drug in the targeted dose, then drug administration by desensitization is an effective and safe option. We present two cases in which we successfully applied nintedanib desensitization with the desensitization protocol we developed.

The first case was a 70-year-old female patient who was diagnosed with IPF and started on nintedanib 150 mg 2 x 1. The patient, who had no problems with the medication for the first 20 days, began reporting urticarial plaques on the face, and upper and lower extremities around 30 minutes after taking the drug on the 21st day. The patient had no history of atopic diseases, such as allergic rhinitis, asthma, atopic dermatitis, urticaria, or of drug hypersensitivity reactions. It was stated that the patient, who was referred by the chest diseases department, should definitely use nintedanib. Mite and pollen sensitivity was detected in the skin test performed with respiratory and food allergens. The skin prick test with nintedanib was negative. Oral desensitization was performed, taking the necessary precautions for the patient, starting at a 1.5 mg dose and increasing every 15 minutes with a total of 150 mg 7-step two

**Table 1:** Nintedanib desensitization protocol

Solution A: 150 mg nintedanib (content of capsule) + 10 cc 0.9% isotonic sodium chloride (Dilution: 1/10, concentration: 15 mg/ml)	Solution B: 1.5 cc A solution + 13.5 cc 0.9% isotonic sodium chloride (Dilution: 1/100, concentration: 1.5 mg/ml)
Step 1	Solution B	1 cc (1.5 mg)
Step 2	Solution B	2 cc (3 mg)
Step 3	Solution B	4 cc (6 mg)
Step 4	Solution B	8 cc (12 mg)
Step 5	SSolution A	1.5 cc (22.5 mg)
Step 6	Solution A	3 cc (45 mg)
Step 7	Solution A	4 cc (60 mg)

concentration (1.5 mg/mL, 15 mg/mL) without premedication (Table 1). Desensitization was completed in 1.5-2 hours and no allergic reaction was observed. Twelve hours later, a full dose of 150 mg nintedanib was given under observation without any problems, and a total daily dose of 300 mg was completed. The second case was a 62-year-old female patient with IPF who was prescribed nintedanib 150 mg 2 x 1. However, since the patient had a previous history of severe anaphylaxis induced by beta-lactam antibiotics, the patient had serious concerns about using this new drug. As with the previous patient, we successfully completed the desensitization protocol. There was no previous case of an allergic reaction to nintedanib in the literature, and no desensitization protocol was indicated for such patients. The desensitization protocol was successfully applied without premedication. Both patients stayed on nintedanib therapy for approximately 10 months without any allergic reaction.

Patients with a history of nintedanib hypersensitivity reaction will be able to receive this treatment safely and effectively with the desensitization protocol we recommend.
